# Continuum of Care and Neurodevelopmental Outcomes in Rural India: An Ambispective Cohort Study

**DOI:** 10.7759/cureus.100544

**Published:** 2026-01-01

**Authors:** Umesh Joshi, Ila Sharma

**Affiliations:** 1 Pediatrics and Neonatology, KD Medical College Hospital and Research Center, Mathura, IND; 2 Pathology and Laboratory Medicine, Government Medical College, Kota, IND

**Keywords:** adherence, developmental outcomes, follow-up programme, neurodevelopmental impairment, outpatient care, prematurity, rural health

## Abstract

Introduction

Neurodevelopmental impairments (NDI) remain a major contributor to long-term disability in children, particularly in rural settings where awareness, early recognition, and continuity of care are limited. Fragmented care-seeking patterns and delayed diagnosis often influence developmental outcomes in these regions. We aimed to compare neurodevelopmental outcomes between children presenting directly to the outpatient department (OPD) and those enrolled in a structured high-risk follow-up programme, and to evaluate the influence of adherence, psychosocial factors, and healthcare access on treatment response.

Methods and material

A total of 73 children with documented perinatal risk factors and mild-moderate NDI were included. Data for the follow-up cohort were retrieved retrospectively from hospital records, whereas OPD children were assessed prospectively. Neurodevelopmental scores (NDS), developmental quotient (DQ)/intelligence quotient (IQ), behavioural profiles, and psychosocial risk factors were recorded. Improvement was measured as a percentage reduction in symptom severity after six months of therapy. Adherence levels and patterns of healthcare access were analysed. Logistic regression with multiple imputation was used to identify predictors of ≥20% improvement.

Results

Baseline NDS did not differ significantly between groups. Children from the follow-up cohort demonstrated more consistent improvement, with 85% achieving a ≥20% reduction in symptoms, compared with 41% of OPD children. The OPD group showed improvement mainly in behavioural symptoms, whereas the follow-up group demonstrated additional gains in IQ and academic performance. Psychosocial adversity, anaemia prevalence, and low adherence were substantially more common in the OPD cohort. Logistic regression did not identify any single independent predictor of improvement, although adherence showed the strongest clinical association.

Conclusions

Children enrolled in structured follow-up programmes achieved more stable and multidimensional neurodevelopmental gains than those presenting directly to OPD care. Poor adherence, psychosocial adversity, and delayed recognition of developmental concerns contributed to weaker outcomes in the OPD group. Strengthening continuum-of-care pathways and improving caregiver awareness are essential for optimising neurodevelopmental outcomes in rural settings.

## Introduction

Neurodevelopmental disorders (NDDs) comprise a broad group of conditions that disrupt cognitive, behavioral, and socio-emotional functioning in childhood. Common disorders such as attention-deficit/hyperactivity disorder (ADHD), autism spectrum disorder (ASD), oppositional defiant disorder (ODD), conduct disorder (CD), and learning disabilities (LD) contribute significantly to academic difficulties, behavioral challenges, and long-term psychosocial impairment. Although global estimates suggest ADHD and AHD affects 5% to 7% of children and approximately 1% to 2%, respectively, the true burden is often underestimated in low- and middle-income settings, where diagnostic delays and limited access to specialized services hinder timely intervention [1,2].

Children born prematurely or with low birth weight (LBW) represent a particularly vulnerable population for NDDs. Perinatal complications, including hypoxic injury, neonatal sepsis, and prolonged neonatal intensive care unit (NICU) admission, adversely influence brain maturation, particularly white matter development and networks involved in attention, executive function, and social communication. These early disruptions frequently manifest later as ADHD, ASD traits, or learning difficulties. Evidence consistently highlights the importance of early detection and structured neurodevelopmental monitoring to mitigate long-term functional impairment [3].

The period immediately following discharge from the NICU offers a critical window for surveillance and intervention, when neuronal plasticity is at its peak. High-risk follow-up programs are designed to capitalize on this opportunity by providing periodic screenings, standardized assessments, caregiver counseling, and timely referrals for therapies. Children enrolled in such programs benefit from early recognition of developmental deviations, improving the likelihood of favorable cognitive and behavioral outcomes [4].

However, in many real-world settings, particularly in resource-constrained regions, not all high-risk infants have access to structured follow-up clinics. A substantial proportion present later to outpatient departments (OPD), often when academic or behavioral problems become apparent. These late-presenting children may experience prolonged diagnostic delays, reduced neuroplastic potential at the time of intervention, and inconsistent engagement with therapy. Sociodemographic factors, such as low parental education, poor awareness of developmental red flags, financial constraints, and rural residence, further exacerbate these disparities, leading to missed opportunities for early developmental support [5].

Comparing outcomes between children receiving structured follow-up and those presenting directly to OPD provides valuable insight into how healthcare access influences developmental trajectories. While these groups are inherently different, they reflect two common pathways through which high-risk children interact with the healthcare system. Understanding these differences can guide policies aimed at strengthening high-risk follow-up frameworks, improving caregiver awareness, and reducing inequities in neurodevelopmental care [6-8].

This study, therefore, investigates neurodevelopmental outcomes in preterm or LBW children across two real-world care pathways: (1) those enrolled in structured high-risk follow-up programs after NICU discharge, and (2) those presenting to OPD services without prior systematic monitoring. By examining differences in adherence to therapy, symptom improvement, socioeconomic influences, and patterns of delayed diagnosis or misrecognition, the study aims to identify gaps in neurodevelopmental care delivery. The findings intend to highlight the need for consistent follow-up strategies and equitable access to early developmental services, particularly for high-risk populations in varied geographic and socioeconomic contexts.

## Materials and methods

This retrospective cohort study was conducted at a single tertiary-care center, KD Medical College Hospital and Research Center (KDMCHRC) in Mathura, Uttar Pradesh, India, from 6th April 2021 to 9th October 2025. The study evaluated neurodevelopmental outcomes in children with a history of NICU admission by comparing two real-world care pathways, namely infants enrolled in structured high-risk follow-up clinics after NICU discharge and children presenting directly to the OPD with neurodevelopmental concerns without previous systematic follow-up. The study design is intended to reflect natural differences in healthcare access rather than equivalence between groups. The study protocol was reviewed and approved by the Institutional Ethics Committee of KDMCHRC (approval No. KDMCHRC/FAC/IEC/2022/134).

Eligible participants included children with mild-to-moderate neurodevelopmental impairment (NDI), ADHD with or without overlapping learning disability, ASD traits, preterm birth or documented LBW, and mild white matter abnormalities (WMA), such as corpus callosum thinning without major structural loss. For children presenting through the OPD pathway, either birth records documenting high-risk delivery or MRI evidence of mild perinatal insult were required. Exclusion criteria included the following: term birth or birth weight greater than 2000 g, severe NDI (greater than two standard deviations below age norms), gross WMA, periventricular hemorrhagic infarction (PVHI), encephalomalacia, bilateral sensory impairments, neuroregression, congenital infections, and major congenital anomalies.

The structured follow-up cohort was derived from a pool of 217 high-risk neonates enrolled in periodic NICU follow-up clinics, of whom 42 children met the inclusion criteria and completed a six-month assessment. The OPD pathway comprised children presenting with neurodevelopmental concerns; 142 children were screened, of whom 22 declined participation, and 41 were lost to follow-up, resulting in a final OPD cohort of 31 children included in the analysis. Analysis of baseline demographics between enrolled and non-enrolled OPD candidates was not feasible due to limited data on those who declined or were lost to follow-up.

Written informed consent was obtained from caregivers of children evaluated through the OPD pathway, while families enrolled in NICU follow-up clinics had previously provided consent for the use of clinical and imaging records as part of institutional follow-up protocols. For children in the structured follow-up pathway, retrospective data were retrieved from existing medical charts, including neuroimaging findings, biochemical profiles, and developmental assessments. For children presenting via the OPD pathway, prospective data were collected using a standardized 10-item questionnaire administered at the time of clinical evaluation. All participants underwent neuroimaging, thyroid function testing, hemogram evaluation, and assessment of vitamin D and vitamin B12 levels.

Neurodevelopmental assessment was conducted using standardized tools. The ADHD symptoms were evaluated using the Vanderbilt ADHD Diagnostic Rating Scale [9], while ASD features were assessed using the Indian Scale for Assessment of Autism (ISAA) [10]. Developmental levels were additionally measured using the Developmental Assessment Scale for Indian Infants (DASII) for children enrolled in NICU follow-up clinics and by developmental quotient (DQ) or intelligence quotient (IQ) testing for children in both pathways [11]. Global developmental evaluation was performed using the Indian Network of Clinical Epidemiology (INCLEN) Diagnostic Tool for Neurodevelopmental Disorders [12].

Primary outcomes included adherence to prescribed therapy, categorized as good or poor, and improvement in neurodevelopmental symptoms at six months, assessed using standardized developmental or behavioral scales. Secondary outcomes also evaluated included the frequency of impulsive or aggressive behaviors, scholastic difficulties, biochemical deficiencies such as vitamin D deficiency, vitamin B12 deficiency, and thyroid abnormalities, as well as the influence of socioeconomic factors and family history. These biochemical parameters were evaluated for clinical screening and exclusion of confounding medical conditions and were not analysed as primary outcome variables.

Statistical analyses were performed using SPSS Statistics version 26.0 (IBM Corp., Armonk, NY, USA). Descriptive statistics included mean, median, and standard deviation for continuous variables. Group comparisons were conducted using chi-square tests for categorical variables, with Fisher’s exact test applied where expected cell frequencies were less than five, and independent t-tests for continuous variables. Multivariable logistic regression analysis was performed to examine the association between structured follow-up and primary outcomes after adjusting for potential confounders such as socioeconomic status and parental education. Subgroup analyses explored differences across gender and socioeconomic strata. Missing data were addressed using multiple imputation for continuous variables and complete-case analysis for categorical variables.

## Results

A total of 73 children fulfilled the study criteria and were included in the final analysis. Of these, 31 (42.5%) first presented to the OPD group, while 42 (57.5%) were enrolled through the structured high-risk follow-up programme (follow-up group). All children had documented perinatal risk factors, and those with severe NDI (INCLEN <30) were excluded from the analysis. Table 1 summarises the baseline demographic and perinatal characteristics of both cohorts, including chronological age at assessment, gestational age at birth, socioeconomic indicators, and initial neurodevelopmental status, demonstrating comparable baseline severity between groups. Baseline neurodevelopmental scores (NDS) ranged from 60 to 95. Approximately one-third (33%) scored between 80-95, one-quarter (25%) between 70-80, and the remaining 42% fell within the 60-70 range. Table 2 presents the baseline clinical diagnostic profile of the two cohorts, including ASD, ADHD, CD, and LD, with overlapping diagnoses permitted. Baseline severity did not differ significantly between the OPD and follow-up groups (p = 0.205).

**Table 1:** Baseline demographic, clinical and socioeconomic characteristics ^†^ p-values derived using independent t-test or Mann–Whitney U test for continuous variables and chi-square or Fisher's exact test for categorical variables, as appropriate. The INCLEN scores represent baseline neurodevelopmental status at first assessment, not outcomes. INCLEN: Indian Network for Clinical Epidemiology; NICU: Neonatal intensive care unit; OPD: Outpatient department; SES: Socioeconomic status

Parameter	Characteristic	Follow-up group (n = 42)	OPD group (n = 31)	Difference/effect size	p-value^†^
Demographics	Age at assessment (months), mean ± SD	70.4 ± 20.1	83.4 ± 24.6	Mean difference: −13.0 (95% CI −23.6 to −2.4)	0.017
	Male sex, n (%)	23 (54.8%)	20 (64.5%)	OR 0.66 (95% CI 0.25–1.75)	0.4
	Rural residence, n (%)	32 (76.2%)	28 (90.3%)	OR 0.35 (95% CI 0.09–1.32)	0.11
Perinatal history	Gestational age at birth (weeks), mean ± SD	32.1 ± 0.7	35.2 ± 1.6	Mean difference: −3.1 (95% CI −3.8 to −2.4)	<0.001
	Birth weight (g), mean ± SD	1384 ± 50	1952 ± 50	Mean difference: −568 (95% CI −591 to −545)	<0.001
	Cesarean delivery, n (%)	24 (57.1%)	7 (22.6%)	OR 4.56 (95% CI 1.62–12.8)	0.004
Clinical risk	NICU admission, n (%)	42 (100%)	26 (83.9%)	—	<0.001
	Hypoxic-ischemic encephalopathy/asphyxia, n (%)	27 (64.3%)	23 (74.2%)	OR 0.63 (95% CI 0.23–1.74)	0.37
Baseline neurodevelopment	INCLEN score (0-100), mean ± SD	72.07 ± 9.45	75.39 ± 9.32	Mean difference: −3.32 (95% CI −8.00 to 1.35)	0.205
	• INCLEN 60-69, n (%)	18 (42.9%)	13 (41.9%)		
	• INCLEN 70-79, n (%)	10 (23.8%)	8 (25.8%)		0.98
	• INCLEN 80-95, n (%)	14 (33.3%)	10 (32.3%)		
Socioeconomic status	Low SES (Kuppuswamy IV-V), n (%)	42 (100%)	26 (83.9%)	—	0.002
	Maternal education ≤8 years, n (%)	30 (71.4%)	7 (22.6%)	OR 8.43 (95% CI 3.00–23.7)	<0.001
Nutritional status	Anemia (Hb <11 g/dL), n (%)	14 (33.3%)	23 (74.1%)	OR 5.75 (95% CI: 2.1–15.7)	<0.001

**Table 2:** Clinical diagnostic profile of the study cohorts *Diagnostic categories are not mutually exclusive; percentages may exceed 100% due to overlapping neurodevelopmental diagnoses. ^†^p-values derived using independent t-test or Mann–Whitney U test for continuous variables and chi-square or Fisher's exact test for categorical variables, as appropriate. The difference in diagnostic prevalence between groups was not statistically significant (p>0.05) ADHD: Attention-deficit/hyperactivity disorder; ASD: Autism spectrum disorder; CD: Conduct disorder; LD: Learning disability; OPD: Outpatient department

Clinical diagnosis	OPD group (n = 31)	Follow-up group (n = 42)	p-value^†^
ADHD with CD*	5 (16.1%)	7 (16.7%)	0.94
LD*	11 (35.5%)	14 (33.3%)	0.84
ASD without ADHD*	12 (38.7%)	15 (35.7%)	0.79
ASD with ADHD*	17 (54.8%)	24 (57.1%)	0.84

**Figure 1:**
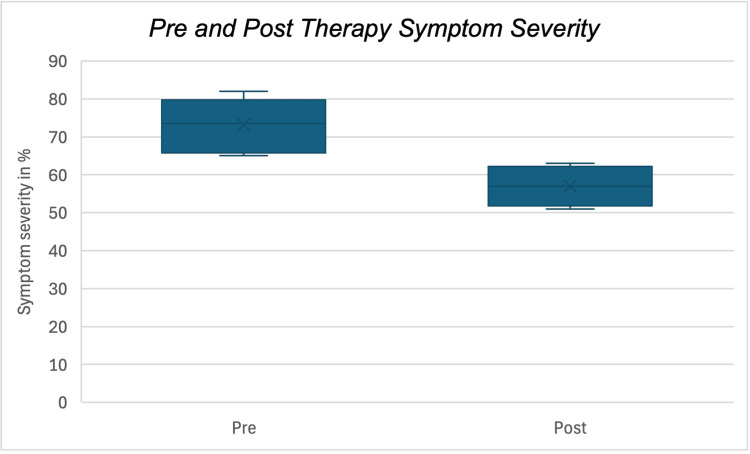
Change in symptom severity over six months

Across the cohort, children demonstrated significant improvement in neurodevelopmental symptom severity after six months of therapy (paired t = 51.07, p < 0.0001). Improvement was assessed as a percentage reduction in neurodevelopmental symptom severity rather than a change in absolute NDS. Reductions in symptom severity ranged between 17% and 25%, reflecting improvements primarily in behavioural regulation, attention, and daily functioning. Boxplots depicting pre- and post-therapy assessment for each group are presented in Figure 1.

Children in the OPD group demonstrated heterogeneous treatment responses. From this group, 17 children (54.8%) achieved ≥20% improvement, 11 (35.5%) achieved 10% to 19% improvement, and the remainder showed <10% change in symptom severity (Table 3). In addition to quantitative symptom reduction, qualitative clinical observations suggested differential patterns of improvement between groups. Children in the OPD cohort primarily demonstrated reductions in overt behavioural symptoms such as hyperactivity and wandering behaviour, with limited observable gains in cognitive or academic functioning during the six-month period. In contrast, children enrolled in structured follow-up more frequently exhibited improvements in attention span, classroom engagement, and functional academic participation, as noted during clinical follow-up visits. These observations were descriptive in nature and were not subjected to formal statistical testing.

**Table 3:** Neurodevelopmental outcomes at the six-month follow-up Improvement was assessed as a percentage reduction in neurodevelopmental symptom severity from baseline. Severity categories are mutually exclusive. The cumulative ≥20% improvement category was derived by summing the ≥25% and 20% to 24% subgroups. The p-values for categorical comparisons were derived using Fisher’s exact test. OPD: Outpatient department; PTM: Parent training model.

Outcome	OPD group (n = 31)	Follow-up group (n = 42)	p-value
Improvement in symptom severity (%)			
≥25% reduction	10 (32.3%)	20 (47.6%)	–
20%-24% reduction	7 (22.6%)	8 (19.0%)	–
10%-19% reduction	14 (45.2%)	14 (33.3%)	–
≥20% reduction (cumulative)	17 (54.8%)	28 (66.7%)	0.338
Parental adherence to the parent training model (PTM)	3 (9.7%)	18 (42.9%)	0.003

**Figure 2:**
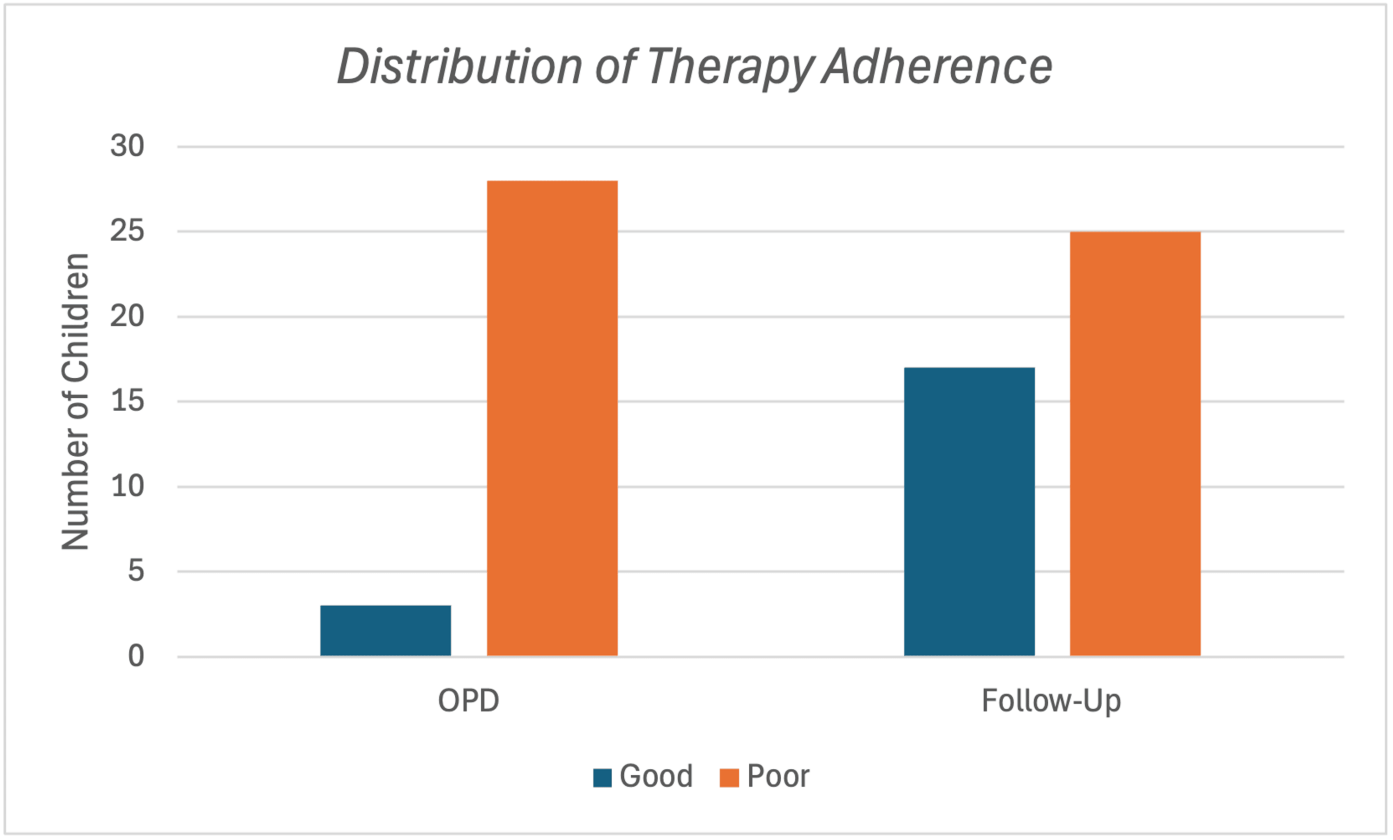
Distribution of therapeutic adherence among OPD children achieving ≥20% symptom improvement OPD: Outpatient department

Psychosocial adversities were markedly more prevalent in the OPD group; specifically, 23 of 31 children (74.2%) reported a history of physical abuse, seven of 31 (22.6%) had a history of early drug exposure, and 23 of 31 (74.2%) displayed wandering behaviour. Only three of 31 parents (9.7%) adhered to the parent training model (PTM), indicating poor therapeutic adherence in this cohort. These contextual risks are summarised in Table 4. Together, these factors appeared to limit the degree of neurodevelopmental progress observed in the OPD group. The distribution of therapeutic adherence among OPD children achieving ≥20% improvement is depicted in Figure 2.

**Table 4:** Psychosocial and behavioural risk factors OPD: Outpatient department

Risk factor	OPD group (n = 31)	Follow-up group (n = 42)	p-value
Physical abuse	23 (74.2%)	2 (4.8%)	<0.001
Drug exposure history	7 (22.6%)	0 (0%)	0.002
Wandering behaviour	23 (74.2%)	5 (11.9%)	<0.001

**Figure 3:**
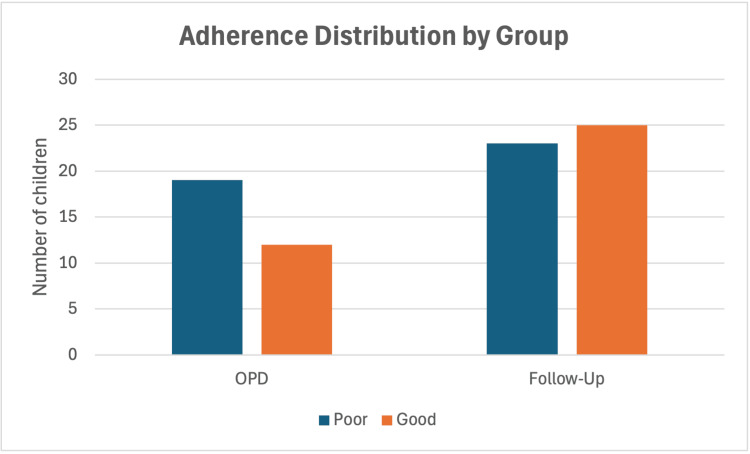
Therapeutic adherence categories within each groups OPD: Outpatient department

Children enrolled in structured follow-up demonstrated more favourable and consistent outcomes. From this group, 28 children (66.7%) achieved ≥20% improvement, while the remainder showed lesser degrees of change in symptom severity (Table 2). Substantial gains were observed across multiple domains, including IQ scores, academic function, and social-communication abilities. The PTM adherence was significantly higher in this group (42%), with families more likely to maintain structured routines and implement behavioural strategies consistently. Although a higher proportion of children in the follow-up group achieved ≥20% improvement compared with the OPD group, this difference did not reach statistical significance (p = 0.338). Qualitative differences in cognitive and academic progress nevertheless favoured the follow-up cohort. Improvements by adherence category are presented in Figure 3.

Therapeutic adherence showed a positive, clinically meaningful association with improvement. Children with good adherence consistently demonstrated greater reductions in symptom burden, although between-group differences (good vs poor) did not reach statistical significance. Both groups independently showed significant pre- to post-treatment improvement in symptom severity (p < 0.0001). Table 5 summarises improvement outcomes across adherence categories.

**Table 5:** Multiple imputation logistic regression (outcome: ≥20% improvement) OPD: Outpatient department

Predictor	β estimate	Odds ratio (OR)	95% CI	p-value
Group (follow-up vs OPD)	−0.061	0.94	0.30-2.93	>0.05
Adherence (good vs poor)	0.761	2.14	0.65-7.04	>0.05
Baseline symptom severity score (per point)	0.044	1.05	0.98-1.11	>0.05

**Figure 4:**
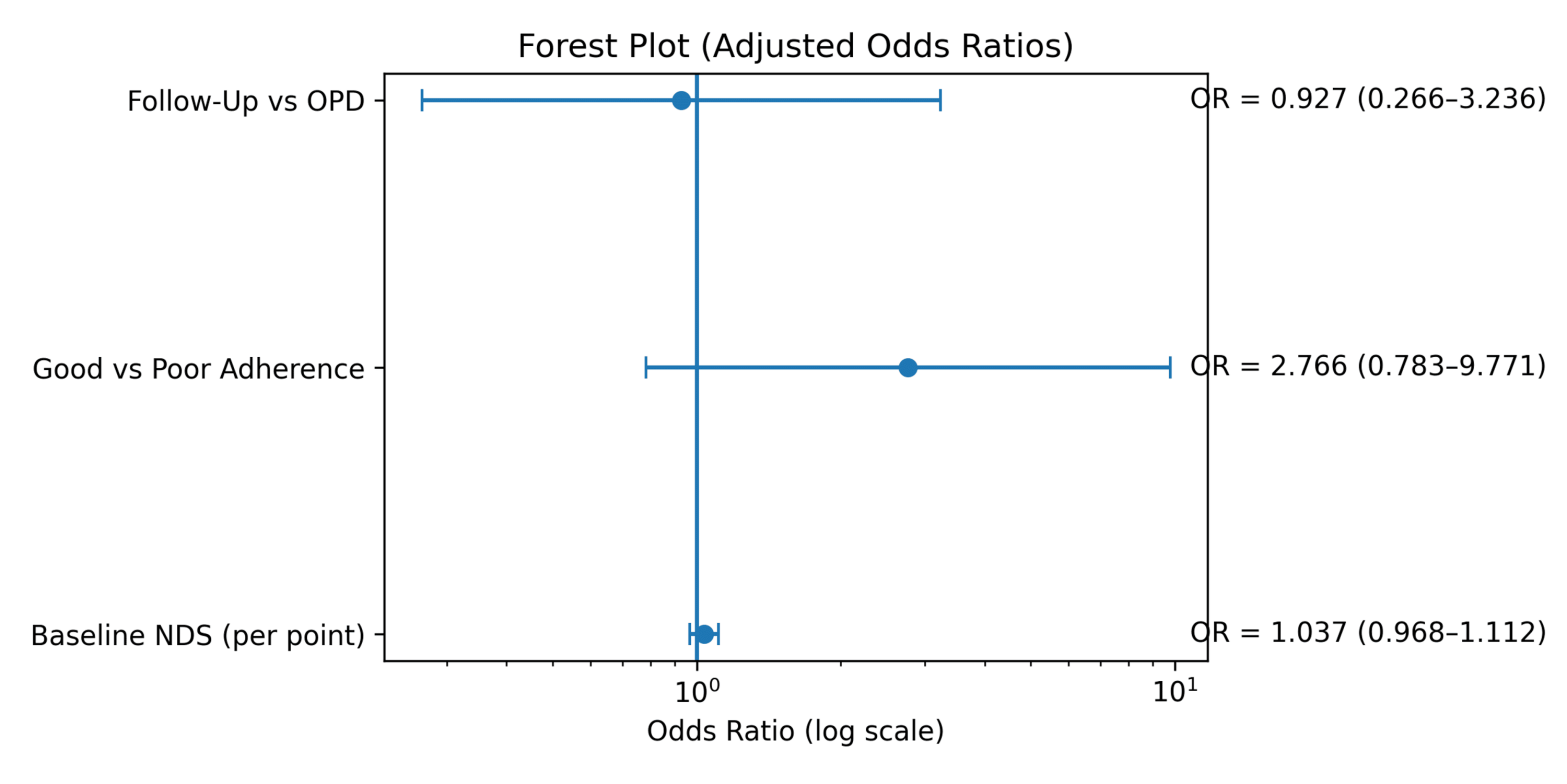
Forest plot of multivariable logistic regression analysis for neurodevelopmental outcome OPD: Outpatient department; NDS: Neurodevelopmental scores

A multivariable logistic regression model was used to identify predictors of ≥20% improvement. Missing data were imputed using five datasets, and estimates were pooled. Predictors included the group category (OPD vs follow-up), therapeutic adherence, and baseline symptom severity score. None of the evaluated variables emerged as statistically significant independent predictors. Follow-up group membership did not significantly increase the odds of achieving ≥20% improvement (OR = 0.94; 95% CI: 0.30-2.93). Good adherence showed the strongest clinical association (OR = 2.14; 95% CI: 0.65-7.04), although confidence intervals crossed unity. The baseline symptom severity score demonstrated a small non-significant effect (OR per point = 1.05; 95% CI: 0.98-1.11). The forest plot of regression predictors is presented in Figure 4, and the complete regression output is summarised in Table 6.

**Table 6:** Logistic model performance

Metric	Value
Area under the curve (discrimination)	0.65
Accuracy	0.77
Precision	0.77
Recall	1.00
F1-score	0.87

Children in structured follow-up exhibited better therapeutic engagement and more consistent behavioural improvement compared with OPD children, while multidomain gains showed a non-significant trend. Psychosocial adversity and poor adherence were major limiting factors among OPD participants. Although no variable independently predicted therapeutic response, adherence showed the strongest clinical association, underscoring its importance in long-term neurodevelopmental care.

## Discussion

Although baseline neurodevelopmental severity was comparable across the OPD and follow-up groups, the OPD cohort demonstrated a substantially higher burden of anaemia, psychosocial adversity, and environmental instability. Together, these factors are known contributors to poorer neurodevelopmental trajectories [13-15]. Importantly, caregiver reports revealed widespread misconceptions about child behaviour and development. In this context, several parents expressed beliefs such as *“thappad maar ke theek ho jaata hai”* ("he improves if we slap him") or reported receiving community advice to seek *“local dawa kar lo, jhaad-phoonk karwa lo”* (local remedies or faith healing). These responses illustrate how culturally entrenched practices may delay recognition of developmental problems. Despite 19 of 31 parents (61.3%) acknowledging that “something was wrong,” many reported reluctance to act due to social stigma, while eight of 31 (25.8%) did not initially accept the diagnosis. Such factors likely shaped the patterns of delayed presentation and fragmented care observed in the OPD group.

Children presenting directly to the OPD showed heterogeneous improvements, with behavioural gains but limited cognitive or academic change. This pattern aligns with research suggesting that intervention benefits are attenuated in the presence of psychosocial adversity [16,17]. Behavioural improvement in the OPD group may also reflect earlier or more frequent use of drugs for externalising symptoms, which can produce rapid behavioural control without corresponding cognitive or academic gains. Factors such as physical abuse (23/31, 74.2%), early drug exposure (7/31, 22.6%), and wandering behaviours (23/31, 74.2%) collectively reflect a high-risk developmental environment. As a consequence, many OPD families reached healthcare through indirect pathways, with 15 of 31 children (48.4%) arriving following a recommendation by a schoolteacher or health worker, and nine of the 31 (29.0%) receiving a developmental diagnosis incidentally during visits for unrelated concerns. These trajectories indicate a lack of structured screening and poor community-level awareness. Qualitative data further highlighted poor consistency of behavioural management at home, and despite this awareness gap, 23 of 31 caregivers (74.2%) recognised the harms of screen-time overuse; most reported difficulty reducing it. Such mismatches between knowledge and implementation further compromised therapeutic effectiveness.

In contrast to the OPD pathway, children in structured follow-up showed more consistent patterns of improvement, particularly in behavioural regulation, with qualitative gains in IQ and academic skills noted during follow-up. These findings are consistent with evidence that early and continuous developmental surveillance is associated with improved long-term outcomes for preterm and high-risk infants [18,19]. Higher PTM adherence (18/42, 42.9%), alongside a lower prevalence of anaemia (14/42, 33.3%), likely facilitated better home reinforcement of therapy. Importantly, caregivers in the follow-up group displayed greater acceptance of the diagnosis and treatment plan, with 16 of 42 parents (38.1%) expressing satisfaction with ongoing therapy, while others were cautiously optimistic. These attitudes reflect the benefit of continuous counselling and predictable follow-up schedules, which likely supported sustained engagement with therapy in structured programmes.

Across both care pathways, adherence emerged as a clinically meaningful factor influencing improvement, consistent with established evidence regarding behavioural parent training and developmental interventions [20,21]. Families in the follow-up pathway demonstrated higher engagement, regular attendance, and better implementation of home strategies, whereas adherence barriers in the OPD cohort appeared strongly linked to stigma, low awareness, and unstructured care-seeking patterns, thereby limiting treatment fidelity. Nearly one-third of OPD families (nine/31, 29.0%) sought care only after comparison with peers, a delay that likely reduced the potential benefit of early intervention. These perceptions weakened sustained engagement and contributed to narrower improvements confined primarily to behavioural symptoms rather than cognitive domains.

Given the heterogeneity of clinical, environmental, and caregiving influences, the logistic regression model did not identify independent predictors of ≥20% improvement. Similar findings have been reported in large cohorts where biological, environmental, and caregiving factors collectively determine outcomes, and no single predictor sufficiently explains developmental trajectories [22,23]. The modest area under the curve observed in this study suggests that improvements cannot be attributed solely to group allocation or adherence but must be interpreted within broader socioeconomic and cultural contexts. Qualitative findings further underscore this complexity, revealing how stigma, delayed presentation, cultural beliefs, and parental uncertainty shape both engagement and outcomes.

Taken together, these findings highlight systemic gaps in the rural continuum of care. Misconceptions about NDS, reliance on punitive methods or faith healing, and delayed recognition due to stigma contribute to late presentation and fragmented management. The fact that 15 of 31 diagnoses (48.4%) emerged through school or health worker referral rather than proactive parental concern mirrors the delayed OPD presentation patterns observed in this study and indicates significant underdiagnosis at the community level. Furthermore, caregivers’ uncertainty about the child’s future and inconsistent implementation of recommended practices, such as screen-time reduction, reflect ongoing educational needs [24,25]. These insights emphasise the critical role of structured follow-up systems in improving awareness, treatment adherence, early identification, and long-term outcomes for children with NDI.

These conclusions, however, must be interpreted in light of certain study limitations. The OPD and follow-up groups differed in psychosocial and environmental risk factors, which may partially explain the observed outcome differences despite similar baseline severity. In addition, differences in data collection methods, with retrospective chart review for the follow-up group and prospective caregiver interviews for the OPD group, may have contributed to differential reporting of psychosocial adversities. The ambispective design reflects routine clinical care, where psychosocial factors are variably present and captured; this heterogeneity represents real-world practice and makes the findings reproducible across similar settings. Variation in assessment tools and limited sample size may have reduced statistical power, and the six-month follow-up period may not reflect long-term neurodevelopmental trajectories, underscoring the need for extended follow-up studies.

## Conclusions

This study suggests that children enrolled in structured high-risk follow-up pathways demonstrate more stable and multidimensional neurodevelopmental progress compared with those receiving unstructured OPD-based care, who predominantly show behaviour-limited gains. These differences underscore the importance of continuity of care, as direct OPD presentation often reflects breaks in the care pathway. Psychosocial adversity and nutritional vulnerabilities were more frequent among OPD children and appeared to limit the extent of improvement despite similar baseline severity. Parental adherence, particularly engagement with PTMs, emerged as an important clinical signal influencing outcomes, even though no variable independently predicted response in multivariable analysis. Importantly, these findings do not imply causality or generalisability but reflect real-world care pathways and caregiver engagement patterns in a rural healthcare setting.
